# Where Can Aluminum Go When Batteries Die?

**DOI:** 10.1002/advs.202518482

**Published:** 2025-11-16

**Authors:** Raymond Kwesi Nutor, Waleed Mohammed, Se‐Ho Kim, Baptiste Gault

**Affiliations:** ^1^ Max‐Planck‐Institute for Sustainable Materials 40237 Düsseldorf Germany; ^2^ Department of Materials Science and Engineering Korea University Seoul 02841 Republic of Korea; ^3^ Department of Materials Imperial College London London SW7 2AZ UK

**Keywords:** aluminum, atom probe tomography, Fe‐alloys, lithium ion‐batteries, sustainability

## Abstract

By 2030, the European Union (EU) is expected to have over 30 million electric vehicles (EVs), making the environmental challenges of end‐of‐life batteries a significant concern. While repurposing extends battery use in less demanding applications, recycling reclaims materials for new products, conserves metal resources, and lessens environmental impact. However, most recycling follows a “shred‐first, sort‐later” approach, which mixes components and makes separation difficult, leading to contamination of recovered materials and reducing their quality and performance. Aluminum (Al), the cathode current collector, is particularly affected by this issue, as impurities build up and have limited solubility, preventing direct reuse in new batteries. In this work, an alternative recycling method is introduced where battery‐derived Al is used as a key alloying element in Fe‐based alloys, fully neutralizing impurity effects. By controlling Al levels and applying thermomechanical processing, typical austenite/ferrite microstructures can be formed with mechanical properties comparable to dual‐phase (DP) and transformation‐induced plasticity (TRIP) steels. Nanoscale characterization shows localized B2 nanoprecipitates in ferrite and impurity segregation at grain and phase boundaries, both influencing fracture behavior. These results demonstrate a scalable and sustainable approach to transforming contaminated battery scrap into a high‐value resource for advanced alloy development.

## Graphical abstract

Battery‐derived aluminum is transformed from contaminated waste into an alloying resource for high‐performance Fe‐based alloys. Through controlled Al content and processing, dual‐phase austenite/ferrite microstructures with TRIP‐like behavior are achieved. Atomic‐scale analysis reveals B2 nanoprecipitation and impurity segregation effects, linking nanoscale features to macroscopic mechanical performance.

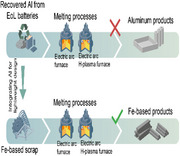

## 1 Introduction

Metals and alloys have been foundational to societal advancement, supporting applications ranging from everyday domestic use to cutting‐edge deep space exploration. At the same time, extracting and producing metals generates a substantial amount of waste and is responsible for ≈40% of all greenhouse gas (GHG) emissions worldwide.^[^ ^1^ ^]^ Now, more than ever, mitigation strategies such as recycling and fossil fuel‐free strategies (e.g., hydrogen and plasma reduction) are essential in reducing the environmental impact of metal production.^[^ ^2^, ^3^ ^]^

As the second most widely used structural metal after steel, Al has broad applications encompassing construction, automotive, and packaging due to its low density (ρ_Al_:2.70 g cm^−3^), high formability, corrosion resistance, and good electrical conductivity.^[^ ^4^, ^5^ ^]^ However, the production of Al from its ores is an energy‐intensive process (≈180 MJ kg^−1^), releasing ≈1.1 billion tons of carbon dioxide (CO_2_) per year and contributing ≈3% of all GHG emissions.^[^ ^6^, ^7^ ^]^ Fortunately, Al has a high recycling rate, with secondary production requiring 90% less energy.^[^ ^5^, ^6^, ^7^ ^]^ However, recycling Al is not without challenges, as impurity contamination is particularly problematic, due to the formation of complex intermetallic compounds that severely degrade the material's properties and hinder processability.^[^ ^5^, ^8^, ^9^, ^10^ ^]^ Mitigating this issue typically involves metal dilution and impurity removal—procedures that are expensive, time‐consuming, and energy‐intensive, thereby contradicting sustainability goals.^[^ ^11^, ^12^ ^]^

In the automotive industry, the growth of EVs presents a looming waste management crisis regarding handling end‐of‐life (EoL) batteries, as highlighted by the European Union's battery regulation.^[^ ^13^ ^]^ EVs are powered by lithium‐ion batteries (LIBs), which typically consist of a cathode, an anode, a separator, an electrolyte, and electrical contacts gathered together in a casing. These components incorporate several important metals, including lithium (Li), cobalt (Co), manganese (Mn), nickel (Ni), copper (Cu), iron (Fe), phosphorus (P), and aluminum (Al), among others.^[^ ^14^, ^15^ ^]^ Al is primarily used as a cathode current collector or as part of the battery casing, typically accounting for a total weight of 30–45 kg per battery pack.^[^ ^16^ ^]^ Thus, recycling this Al and the other strategic battery materials strengthens the EV supply chain.^[^ ^17^, ^18^ ^]^

In reality, though, battery recycling remains a complex, expensive, and technically demanding process, largely due to the wide variety of battery chemistries and the distinct physicochemical properties of the components.^[^ ^18^ ^]^ Furthermore, hazardous constituents like halogens and polymers in batteries can release toxic pollutants such as hydrogen fluoride and dioxins during recycling, raising concerns about the true environmental and economic value of battery recycling.^[^ ^14^, ^17^ ^]^

For Al recovery, the cathode material is the primary source of contamination, which, depending on the battery type, can include Ni, Li, Mn, P, Co, along with Fe, Si, and Cu from other components.^[^ ^17^ ^]^ Impurity elements like Ni, Co, and Fe enhance the formation of intermetallic compounds because their solubility in Al is very low (<0.1 wt.%), which negatively affects formability and mechanical properties.^[^ ^19^, ^20^, ^21^ ^]^ In contrast, Cu, Mn, and Si are the main alloying elements of the 2xxx, 3xxx, and 4xxx series, respectively, and contribute to strengthening via the formation of precipitates, particles, or dispersoids.^[^ ^22^, ^23^ ^]^ Oxygen (O), meanwhile, poses the greatest threat to the recyclability of Al from EoL batteries, since it is known that alumina (Al_2_O_3_) forms on Al current collectors during battery operation due to reactions with residual moisture or electrolytes.^[^ ^24^ ^]^ The formation of Al_2_O_3_ on the Al current collector inhibits interfacial wetting and fusion, posing challenges for downstream recycling operations such as mechanical separation, leaching, and remelting. These issues will render the recovered Al undesirable for high‐purity applications, like in batteries and microelectronics.

Heavily contaminated Al can be repurposed for other applications (i.e., upcycling or downcycling) to augment the Al supply chain. Aluminum's extensive use across multiple alloy systems—driven largely by the need for lightweight solutions—creates a strong incentive to boost recycled content in alloy manufacturing, paving the way for more sustainable production in the years ahead.^[^ ^3^ ^]^ In steels, Al is often used to stabilize the ferrite phase, improve precipitation hardening (e.g., through B2‐phase formation), lower overall alloy density, and act as a deoxidizer during melt processing.^[^ ^25^, ^26^, ^27^ ^]^ Al plays a key role in forming the coherent γ′ (Ni_3_Al) precipitate, which is essential for providing excellent creep resistance and high‐temperature stability of nickel‐based superalloys.^[^ ^28^ ^]^ Similarly, in titanium alloys, Al is a strong α‐phase (HCP) stabilizer, significantly enhancing solid‐solution strengthening while also reducing alloy density.^[^ ^29^ ^]^ Al has also been employed to promote body‐centered cubic (BCC) phase formation in emerging high‐entropy alloys (HEAs), which are also referred to as compositionally complex alloys (CCAs) or multi‐principal element alloys (MPEAs).^[^ ^30^ ^]^ Of the alloy systems discussed, Fe‐based alloys offer greater compositional flexibility, providing more robust impurity tolerance to offset the Fe, Mn, Ni, and Co contamination issues with battery recycling.

Given these complexities, several key questions emerge in the context of Al recycling from EoL batteries. To address these questions, we employed a two fold approach aimed at augmenting the Al supply chain either in the Al alloy class or as an alloying element in other systems. The first part of the work targeted the feasibility of using hydrogen plasma reduction technology to “clean up” Al scrap for direct reuse. This is because hydrogen plasma reduction offers a sustainable alternative for metal production, where complex oxides derived from both native ores and waste materials are concurrently melted and reduced in an electric arc furnace (EAF).^[^ ^31^, ^32^ ^]^ In a recent example, Jovičević–Klug et al.,^[^ ^33^ ^]^ demonstrated that metallic Fe can be obtained from red mud (a waste material from bauxite refinement) through the exploitation of density and viscosity differences to separate metal from oxides. The second part of the work then demonstrates integration of recovered Al scrap from EoL batteries with Fe‐based alloy scrap sourced from archived materials of previously completed projects. Hereafter, micro‐to‐nanoscale characterization techniques, including scanning electron microscope (SEM), energy‐dispersive X‐ray spectroscopy (EDS), electron backscatter diffraction (EBSD), and atom probe tomography (APT), were used for phase and compositional analysis of the studied samples. Our results indicate that the recovered Al is unsuitable for direct reuse in Al alloys due to severe oxide contamination; however, it can serve as a valuable feedstock for Fe‐based alloys. Collectively, these findings establish a reference framework for incorporating multiple scrap streams into the design of next‐generation impurity‐tolerant alloys.

## 2 Results

### 2.1 Characterization of Al Scrap

**Figure 1:**
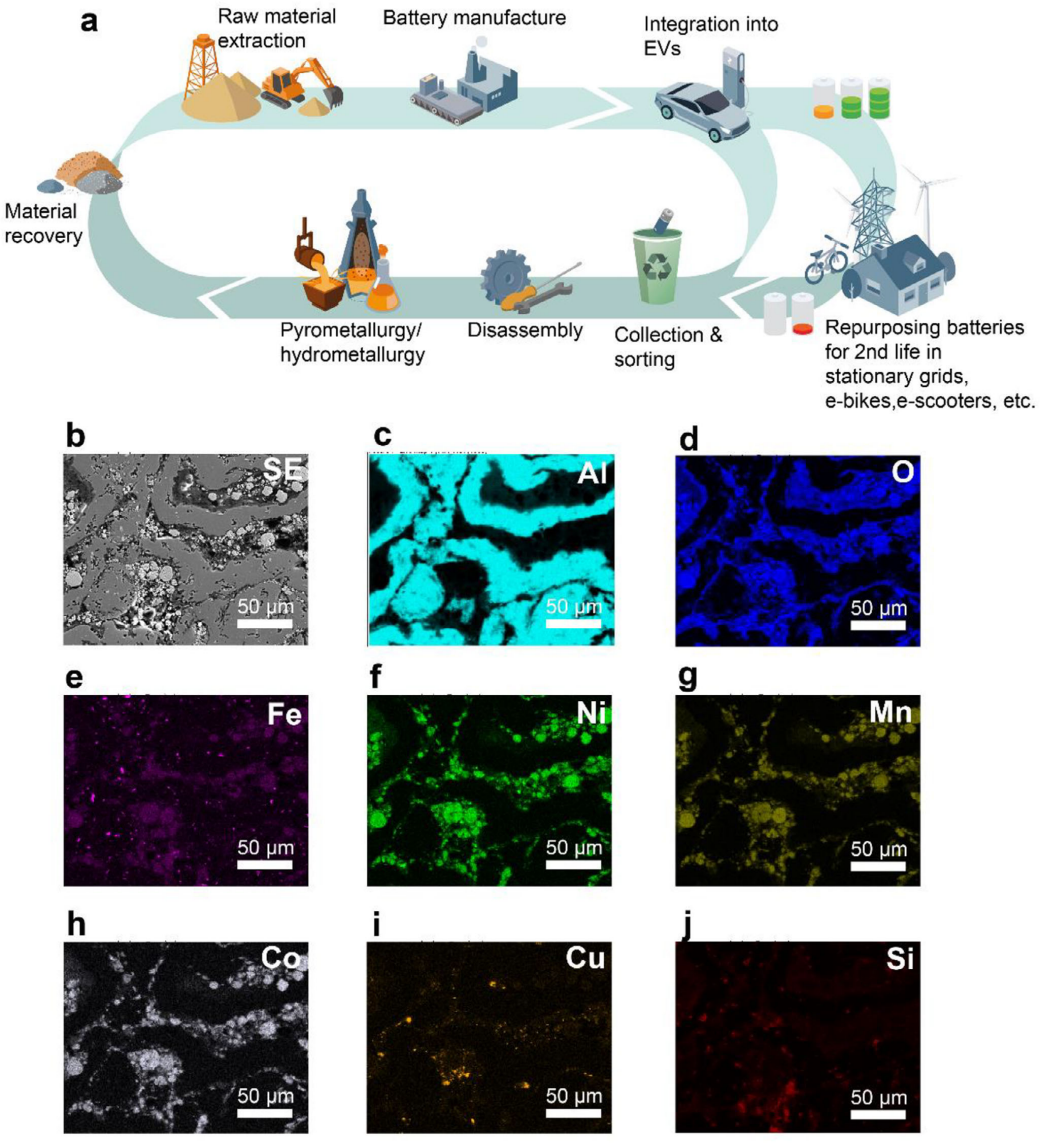
Outlook for the current recycling landscape of battery materials from electric vehicles. a) Schematic representation of the circular economy for lithium‐ion batteries (LIBs), illustrating the flow from raw material extraction to end‐of‐life (EoL) recovery. b) Secondary electron SEM micrograph of Al shreds recovered from an electric vehicle (EV) battery scrap. c–j) Corresponding EDS elemental maps showing the distribution of Al, O, Fe, Ni, Mn, Co, Cu, and Si, respectively.

An overview of the LIB lifecycle, from raw material extraction to EoL, is depicted in **Figure** **1**a. The constituent metals are extracted and refined at geographically dispersed mining sites before being assembled into battery cells. Following assembly, the batteries are incorporated into EVs for their primary use phase. Production scraps are typically preferred for direct recycling, as they are free from contamination arising from electrochemical cycling.^[^ ^34^ ^]^ In cases where the full capacity of a battery is not depleted, repurposing into secondary applications such as stationary storage or light electric mobility can significantly extend its functional lifetime.^[^ ^35^ ^]^ EoL batteries are collected from diverse sources and subsequently sorted according to their chemical composition and physical condition to facilitate efficient recycling.^[^ ^36^ ^]^

Before dismantling, the batteries are fully discharged to eliminate potential electrical hazards.^[^ ^18^ ^]^ Following dismantling, the batteries undergo size‐reduction processes such as crushing and shredding, which facilitate the separation of materials based on differences in density, magnetism, or morphology. Ferromagnetic materials, primarily from battery casings, are recovered through magnetic separation, while selective metal extraction is achieved using either hydrometallurgical or pyrometallurgical methods.^[^ ^17^ ^]^ Recovered metals are then reused in battery production if they meet quality standards; otherwise, they are refined to meet industrial requirements, reducing reliance on newly mined resources. In this study, Al scrap was obtained via mechanical separation—comprising shredding and density‐based sorting—of various EoL nickel–manganese–cobalt oxide (NMC)‐based LIBs, as carried out by the industrial partner, Eramet. Figure 1b–j shows the SEM micrograph and corresponding EDS elemental maps of the analyzed Al scrap. The dark grey areas in the SEM image are attributed to the Al shreds, while the brighter, spherical features correspond to residual NMC oxide particles, primarily enriched in Ni, Mn, Co, and O. Regions with localized enrichment of Fe, Cu, and Si are also observed.

### 2.2 Plasma Melting of Al Scrap

Digital images of the solidified Al ingot after hydrogen plasma reduction are shown in Figure S2a,b (Supporting Information). The resulting ingot is brittle and lacks structural integrity, displaying a loosely consolidated structure that points to insufficient melting during processing. Moreover, surface porosities are seen on the ingot, likely originating from trapped gases during the melting.^[^ ^32^ ^]^ SEM analysis (Figure S2c, Supporting Information), along with the corresponding EDS elemental maps, reveals the presence of large, needle‐like structures within the Al matrix that correspond to oxygen‐enriched regions. These oxide inclusions are found to be alumina (Al_2_O_3_), which form when oxygen‐contaminated aluminum is melted and usually impair melt wettability and fluidity.^[^ ^37^ ^]^ The Al matrix also hosts intermetallic phases of different morphologies: blocky grey (Mn‐rich), white platelets (Cu‐rich), and grey Chinese‐script (Ni‐rich) (Figure S3a, Supporting Information). EBSD analysis, with representative indexed patterns shown in Figure S3b–d (Supporting Information), confirmed these phases as µ‐Al_4_Mn (hexagonal), θ‐Al_2_Cu (tetragonal), and τ‐Al_9_(Fe,Ni)_2_ (monoclinic), respectively.^[^ ^38^, ^39^, ^40^ ^]^ The intermetallic phases, in combination with micrometer‐sized Al_2_O_3_ platelets, are expected to impose considerable challenges on the processability of Al.

Hydrogen plasma, while effective for reducing many metal oxides,^[^ ^41^ ^]^ was unable to yield high‐purity Al from battery scrap. Despite the high temperatures attainable by plasma species, the reduction of Al_2_O_3_ remains thermodynamically and kinetically constrained due to the exceptional thermodynamic stability of alumina, poor thermal conductivity of oxide particles, limited plasma–surface mass transport, and the rapid formation of an alumina shell that blocks hydrogen diffusion to the reaction interface.^[^ ^42^, ^43^ ^]^ This results in incomplete reduction, heterogeneous melting, and an extremely brittle end product unsuitable for further processing. Ultimately, the plasma‐melted Al falls short of the purity and processability standards required for reuse. As such, solid‐state recycling methods like hot extrusion and friction stir processing are viable options.^[^ ^44^ ^]^ Alternatively, the recovered Al can be downcycled or integrated into other alloy classes that have a higher tolerance for the impurities.

### 2.3 Al Scrap Integration

Beyond the conventional Al‐alloys, Al is a potent alloying element across several other metallurgical systems. Hence, integrating heavily contaminated Al into alternative alloy classes presents a practical strategy to supplement and diversify the Al supply chain. We demonstrate this strategy by combining the recovered Al scrap from EoL batteries with Fe‐based alloy scrap sourced from archived materials from previously completed projects. We designed two alloy compositions by adding 8 wt.% and 12 wt.% Al scrap to the Fe scrap, denoted as 8‐SCA and 12‐SCA (i.e., scrap alloy), respectively. The Al contents of 8 wt.% and 12 wt.% were selected based on the Fe‐Al equilibrium phase diagram since α‐Fe dissolves up to ≈12.5 wt.% Al in the A2 (BCC) phase at high temperatures (≥900 °C).^[^ ^27^ ^]^ Accordingly, 8‐SCA represents an intermediate solubility composition, while 12‐SCA corresponds to the upper limit. **Table** **1** shows the chemical composition of all the samples.

**Table 1:** Chemical compositions in wt.% of the studied samples obtained from ICP‐OES analysis.

Sample	Al	Co	Cr	C	Cu	Fe	Li	Mn	Ni	Si	S	V
Al scrap	Bal.	1.24	<0.01	3.23	2.70	0.46	0.72	1.90	2.35	0.12	‐	‐
Fe‐scrap	‐	‐	12.9	0.12	‐	Bal.	‐	29.6	11.8	‐	‐	‐
8‐SCA	4.59	0.24	6.09	0.06	0.12	Bal.	‐	12.9	6.05	0.06	<0.01	0.18
12‐SCA	7.10	0.10	5.89	0.22	0.14	Bal.	‐	12.5	4.93	0.08	<0.02	<0.01

### 2.4 Microstructural Analysis of SCA Samples

#### 2.4.1 Bulk and Microscale Analysis

**Figure 2:**
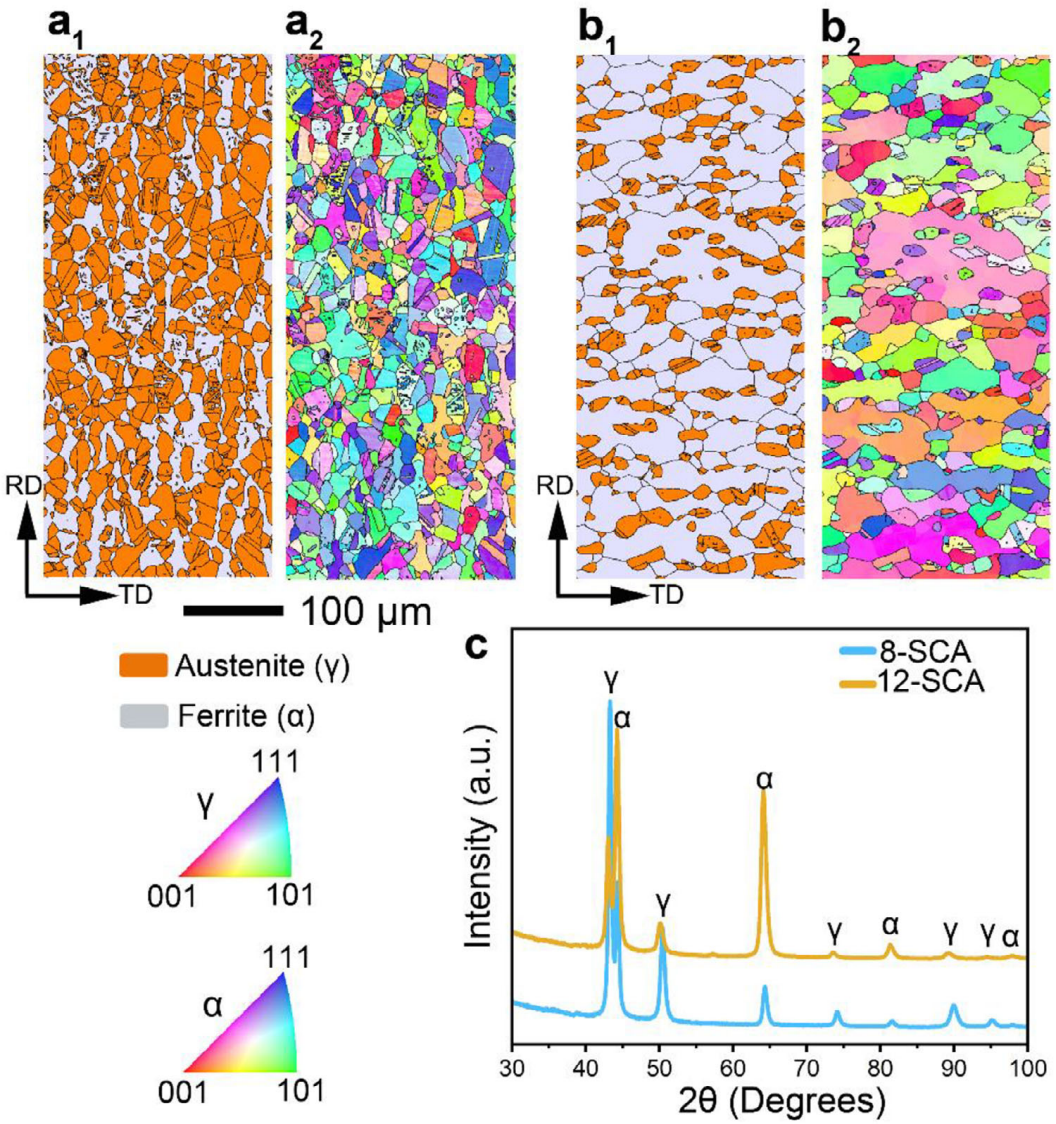
Microstructural and phase analysis of the SCA samples. EBSD phase maps of the **a_1_**) 8‐SCA and **b_1_**) 12‐SCA samples. Inverse pole figure (IPF) maps of the **a_2_**) 8‐SCA and **b_2_**) 12‐SCA samples. (black lines represent grain boundaries, TD = transverse direction; RD = rolling direction). c) XRD patterns of the studied SCA samples.

Table 1 summarizes the chemical compositions of the samples, determined using ICP‐OES. As expected, the 12‐SCA sample (13.6 at.%) contains more Al than the 8‐SCA sample (9 at.%). The equilibrium phase diagrams (Figure S1, Supporting Information) suggest that both samples (annealed at 1000 °C and water‐quenched) should possess dual‐phase austenite (γ)/ferrite (α) microstructures. This prediction is later confirmed by electron backscatter diffraction (EBSD) phase mapping and the X‐ray diffraction (XRD) patterns (**Figure** **2**). The α volume fraction in the 8‐SCA sample is substantially lower (≈27%) compared to the 12‐SCA sample (≈75%). The dominant γ matrix indicates that the 8‐SCA sample is austenite‐based duplex steel, whereas the 12‐SCA sample is a ferrite‐based duplex steel, characterized by its major α fraction. The observed transition aligns well with previous findings since Al is a strong ferrite stabilizer.^[^ ^27^, ^45^ ^]^ The inverse pole figure (IPF) maps (Figure 2a2,b_2_) show that both the γ and α grains are fully recrystallized with a near‐random orientation. Figure S4 (Supporting Information) reveals that the average γ and α grain sizes in the 8‐SCA sample are ≈31.1 and 29.8 µm, respectively, whereas the 12‐SCA sample exhibits average γ and α grain sizes of ≈26.8 and ≈64.8 µm, respectively.

#### 2.4.2 Nanoscale Compositional Analysis

**Figure 3:**
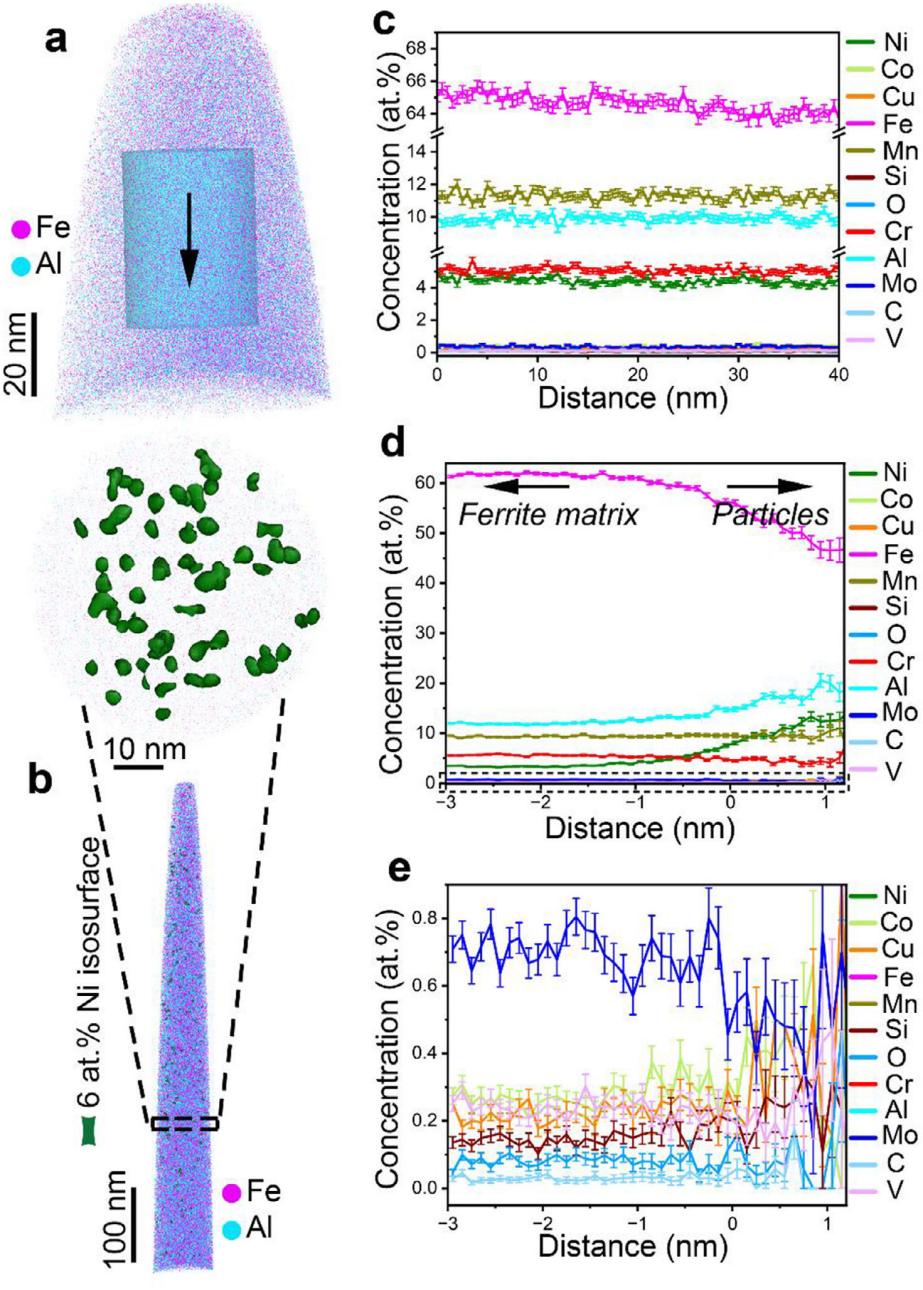
Elemental distribution within the γ and α phases in the recycled 8‐SCA sample. a) 3D APT reconstruction of the grain interior within the γ phase. b) 3D APT reconstruction highlighting a 6 at.% Ni isosurface, with an outlined region revealing nanoparticle distribution within the α phase. c) 1D concentration profile of constituent elements across the γ grain interior, extracted along the cylindrical analysis volume. d) 1D proximity histogram demonstrating the compositional changes between the α matrix and particles. e) Corresponding magnified view of the region marked by the dashed rectangle in (d).

To investigate the chemistry of the alloys, we first performed APT liftouts from the individual phases. **Figure** **3**a,b shows the APT reconstructions of the γ and α phases in the 8‐SCA sample. The corresponding concentration line profiles (Figure 3c) indicate that the γ phase is chemically homogeneous, with no significant elemental segregation observed. The γ phase consists of ≈11.5 at.% Mn, 9.8 at.% Al, 5.3 at.% Cr and 4.3 at.% Ni, while the α phase contains ≈9.2 at.% Mn, 11.9 at.% Al, 5.8 at.% Cr and 3.2 at.% Ni. The notable enrichments of Mn and Ni in the γ phase and Al and Cr in the α phase are also confirmed by the SEM‐EDS analysis in Figure S5 (Supporting Information). The increased amounts of Mn and Ni in the γ phase and Al and Cr in the α phase are consistent with their respective roles as austenite‐ and ferrite‐stabilizing elements.^[^ ^25^, ^26^ ^]^ The α phase also consists of near‐spherical nanoprecipitates (with an average particle size of 3.26 ± 0.04 nm and number density of 5.95 ×  10^22^ m^−3^), as highlighted by the 6 at.% Ni isosurface. Proximity histograms (Figure 3d,e) indicate increased concentrations of Ni (+10.4 at.%) and Al (+6.2 at.%) in the precipitates relative to the α matrix. Trace element analyses show that Mo is rejected from the α matrix, while the concentrations of C, O, V, and Si remain similar between the α matrix and the precipitates. Based on their compositional stoichiometry and shape, these nanoparticles are consistent with (Fe,Ni)Al B2‐precipitates.^[^ ^46^ ^]^

APT analyses demonstrate that the 12‐SCA sample exhibits a similar chemical profile, i.e., a chemically homogeneous γ phase, and an α phase containing uniformly dispersed NiAl‐rich nanoprecipitates (Figure S6, Supporting Information). Compared to the lower Al sample (8‐SCA), these nanoprecipitates in the α phase coarsen to an average size of 8.17 ± 0.26 nm with a number density of 1.83 ×  10^23^ m^−3^. The B2 phase is an intrinsically hard intermetallic compound that forms by ordering of α‐Fe at Al concentrations of ≈10–32 wt.%, and is known to reduce room‐temperature ductility.^[^ ^27^, ^46^ ^]^ These ferrite localized B2 precipitates typically generate stress concentrations due to massive dislocation restriction in the phase, especially when they increase in size and number fraction. Additional SEM‐EDS mapping (Figure S7, Supporting Information) reveals the presence of Cr‐rich particles at the ferrite GBs and grain interior. These Cr‐rich particles are also expected to further embrittle the ferrite phase and the 12‐SCA sample as a whole.

**Figure 4:**
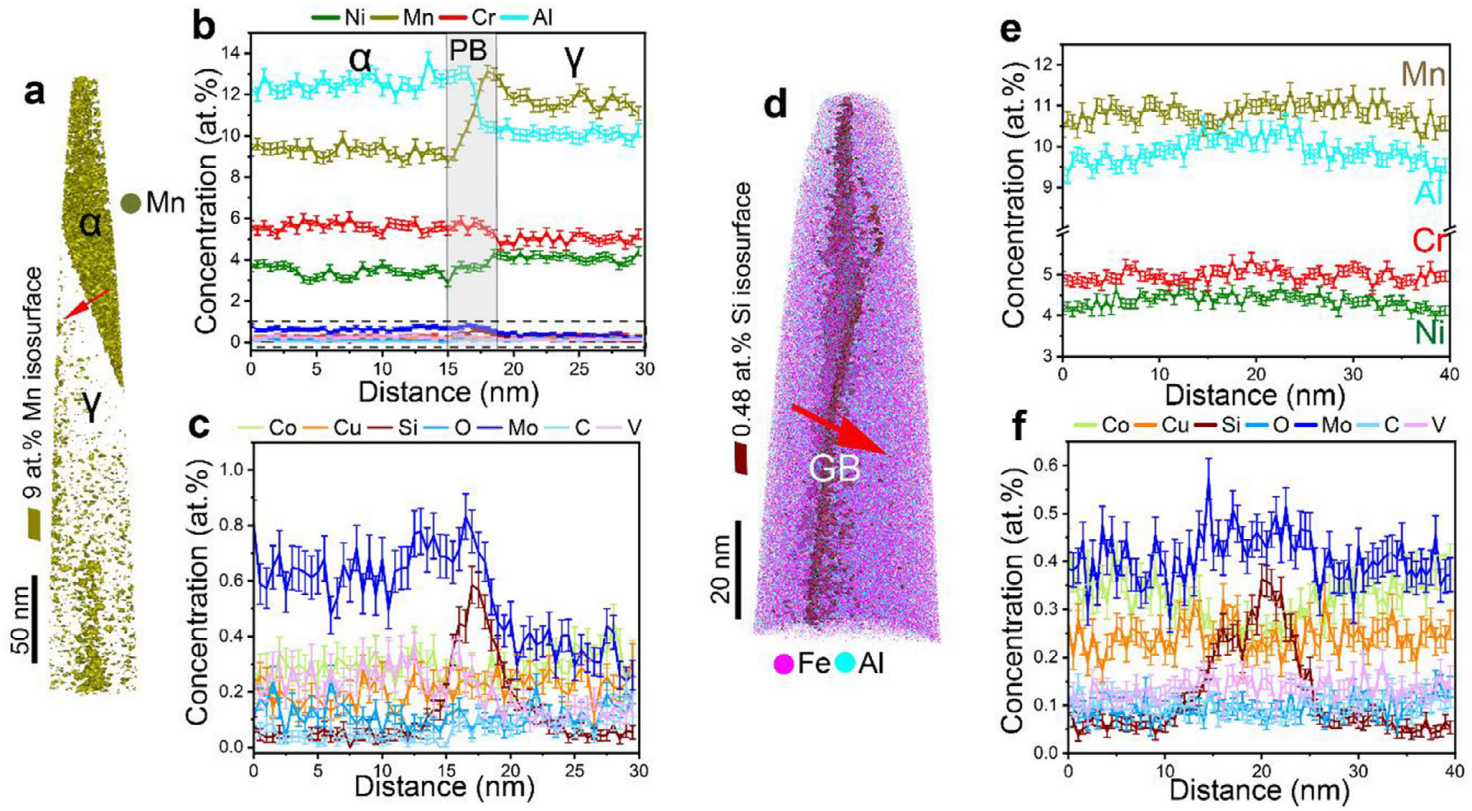
Segregation tendencies at the phase and grain boundaries in the recycled 8‐SCA sample. a) 3D APT reconstruction with a 9 at.% Mn isosurface showing the γ/α interface. b) 1D concentration profile across the γ/α phase boundary. c) Corresponding magnified view of 1D concentration scan of impurity elements from the highlighted region in (b). d) 3D APT reconstruction with a 0.48 at.% isosurface revealing a grain boundary (GB) in the γ matrix. e) 1D concentration profile across the GB, along with f) a magnified view showing the distribution of impurities across the GB.

Interfacial segregation of solute and impurity atoms ensures a system's free energy minimization.^[^ ^47^ ^]^ Nonetheless, segregation of solute atoms to interfaces like phase and grain boundaries can compromise interface cohesion, thereby promoting fracture.^[^ ^48^, ^49^ ^]^ As such, understanding the impacts of segregation is important for recycle‐friendly or impurity‐tolerant alloy design. **Figure** **4**a shows the 3D APT reconstruction across a γ/α interface, delineated by a 9 at.% Mn isosurface in the 8‐SCA sample. Figure 4b,c reveals that significant elemental partitioning occurs for Mn, Al, Ni, Cr, and Mo in the austenite and ferrite phases. A slight increase of Mn is detected on the austenitic side of the γ/α interface, with a concentration of ≈13.1 at.% vs ≈11.6 at.% in the austenite matrix. An enrichment of Si, reaching a maximum of ≈0.58 at.%, is obvious at the γ/α phase boundary. In contrast, no appreciable segregation of other impurity elements, such as Co, Cu, C, Mo, O, and V, is detected at the interface. The modest Mn enrichment and profound Si segregation are believed to occur due to the incomplete phase transformation and the interaction between solute atoms with vacancies during annealing and cooling.^[^ ^50^ ^]^

Using a 0.48 at.% Si isosurface, we visualize a grain boundary (GB) in an austenite grain (Figure 4d). 1D concentration profiles extracted across the GB (Figure 4e,f) reveal pronounced Si segregation, with a peak value of ≈0.38 at.%, whereas a slight Mn depletion is seen at the GB (≈10.5 at.%). Meanwhile, the other solute elements are relatively uniform across the boundary region. The observed interfacial segregation of Mn and Si has been reported to weaken boundary strength, which is detrimental to fracture resistance.^[^ ^51^ ^]^ Next, we examine the tensile properties of the studied samples.

### 2.5 Room Temperature Tensile Properties

**Figure 5:**
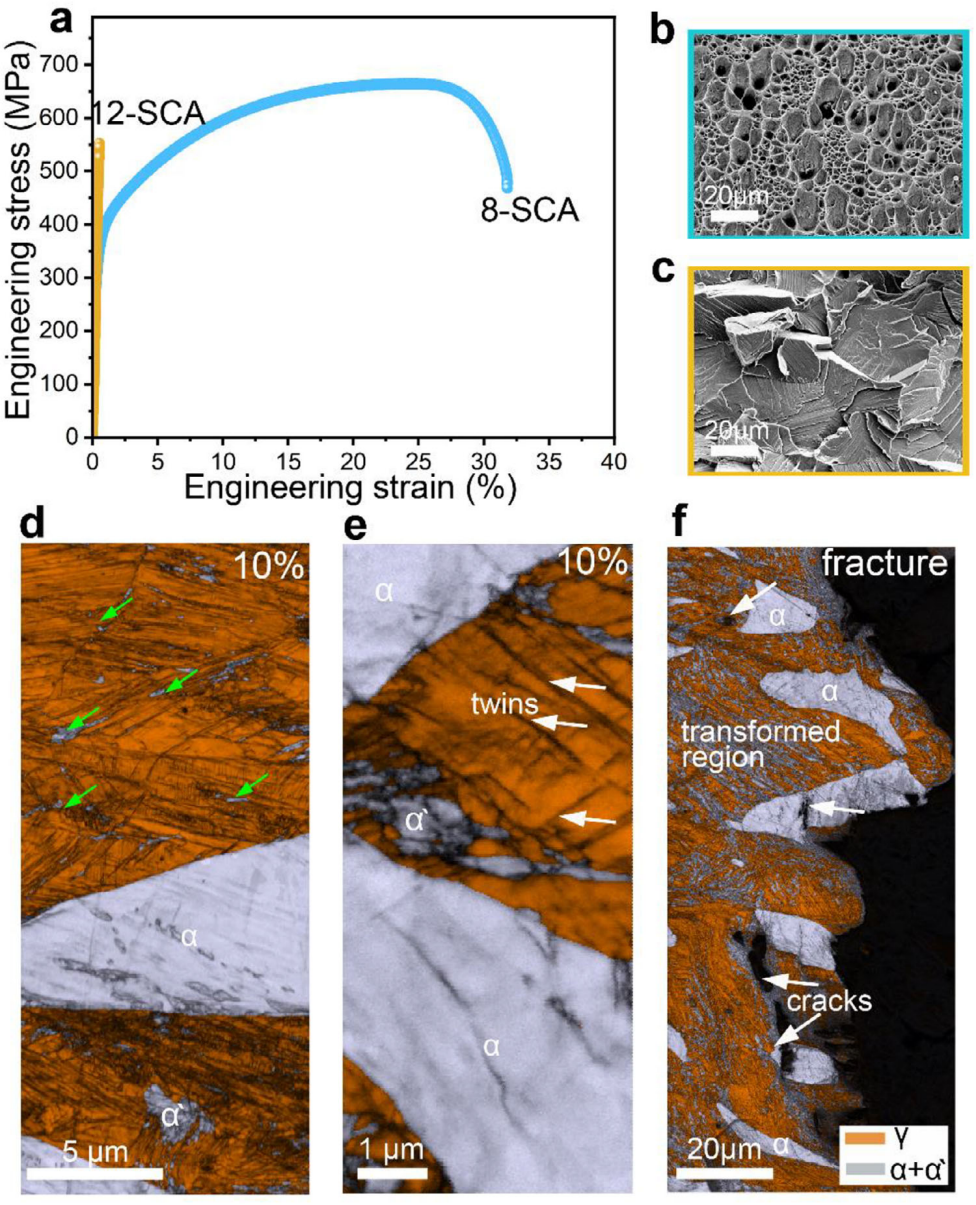
Room temperature tensile performance and deformation mechanisms of the studied SCAs. a) Tensile stress–strain curves of the SCA samples. SEM fracture morphologies of the b) 8‐SCA and c) 12‐SCA alloy specimens. EBSD IQ + phase maps at d,e) ≈10% strain showing martensite nucleation sites, and f) the fracture region of the 8‐SCA sample.

The engineering stress–strain curves in **Figure** **5**a represent the room‐temperature tensile properties of the recycled SCA samples. The 8‐SCA sample demonstrates a yield strength (YS) of 407 ± 6 MPa, an ultimate tensile strength (UTS) of 665 ± 12 MPa, and a fracture elongation of 32 ± 2 MPa. In contrast, the YS rises to 530 ± 4 MPa in 12‐SCA; however, this enhancement is accompanied by pronounced embrittlement, as evidenced by a drastic reduction in fracture elongation to 0.52 ± 0.1 MPa. We note that the observed tensile properties of the SCA samples are comparable to conventional Fe‐Al alloys with similar Al contents, dual‐phase, TRIP, and high‐strength low‐alloyed (HSLA) steels.^[^ ^52^, ^53^ ^]^ Nevertheless, considering the wide microstructural variability in the SCA phase diagram (Figure S1, Supporting Information), tailored thermomechanical processing can be used to exploit interactions between principal elements and residual impurities to optimize performance. Figure 5b,c show the fracture surfaces of the tensile‐tested samples. The 8‐SCA sample reveals a uniformly dimpled fracture surface, indicative of extensive micro‐void coalescence and ductile fracture, while the 12‐SCA alloy exhibits pronounced cleavage facets and well‐defined river patterns, reflecting a brittle fracture.^[^ ^54^ ^]^ This indicates that the austenite‐ferrite ratio, which is dependent on the Al amount, controls this ductile‐to‐brittle transition.

The deformation microstructure near the fracture surface was analyzed to understand the deformation mechanisms. Figure 5d,f shows the EBSD phase map overlaid with the image quality (IQ) map of the deformed 8‐SCA sample. Since EBSD analysis relies on crystallographic symmetry, distinguishing between the bcc‐α and body‐centered tetragonal (bct) α′‐martensite phases is inherently challenging due to their closely related lattice structures.^[^ ^55^ ^]^ Consequently, both phases are indexed as bcc in the EBSD phase maps. A small fraction of nanoscale α′‐martensite, indicated by green arrows, is seen along GBs, twin boundaries, and at the γ/α interfaces at ≈10% tensile strain. These defect sites are characteristic nucleation sites for martensite due to the high stress concentrations at these regions.^[^ ^56^, ^57^, ^58^, ^59^, ^60^ ^]^

Another indication of the martensitic transformation is the significant decrease in the γ phase fraction. It drops from ≈73% in the undeformed state to 35% near the fracture zone (see Figure S8, Supporting Information). We note that this α′‐martensite is limited to the γ phase (indicative of a metastable austenite), while no martensite is observed in the α‐ferrite. We also did not detect any intermediate hcp ε‐martensite for the γ→α′ transformation, as a high localized strain is seen in the transformed austenite plus martensite regions, as seen by the EBSD kernel average misorientation (KAM) maps (see Figure S9, Supporting Information). As an indicator of strain distribution, the KAM is proportional to the density of geometrically necessary dislocations (GNDs).^[^ ^61^ ^]^ With an average KAM of ≈2.21° in the γ phase vs ≈1.65° in the α+α′ region, the KAM maps reveal a clear plastic deformation mismatch between the constituent phases.

The B2 precipitates in ferrite lead to dissociated superlattice dislocations, suppressing dislocation glide and cross‐slip, which in turn embrittles the ferritic regions.^[^ ^62^ ^]^ While the nanoscale B2 particles provide sufficient hardening of the α‐Fe matrix, they can also generate local lattice strain and heterogeneous stress fields.^[^ ^27^ ^]^ Such strain localization facilitates microcrack initiation under tensile loading, evident from the pronounced microcracks at the phase interfaces and within the ferrite (see Figure 5f). Overall, given the γ/α duplex microstructure, the plastic strain distribution during tensile loading is inherently heterogeneous, with the softer γ phase accommodating larger local strains.^[^ ^63^ ^]^ This promotes strain‐induced martensitic transformation (TRIP effect), whereby metastable γ regions progressively transform to α′, enhancing work hardening and delaying plastic instability.^[^ ^64^, ^65^ ^]^ While the γ‐austenite is generally tough, the transformed α′‐martensite and the α‐ferrite (together with the B2 precipitates) are rather hard. Thus, a large strain incompatibility is created at the γ/α, α/α′, and γ/α′ interfaces, making them viable sites for void nucleation.^[^ ^66^, ^67^ ^]^ Beyond stress localization, the segregation of particular impurity elements undermines the cohesive strength of the interface.^[^ ^48^, ^51^ ^]^ Extremely harmful decohesion elements, such as P and S, were not detected throughout the specimen, indicating the scrap sources are devoid of these elements. Nonetheless, the Mn and Si segregation at γ/α interfaces and GBs could diminish the interfacial cohesion, hence making it more prone to void nucleation.^[^ ^50^ ^]^

In the case of the 12‐SCA sample, we did not observe any martensitic transformation since the austenite fraction remains relatively constant (≈25%) after deformation. The 12‐SCA sample exhibits high brittleness, with no plastic deformation zone (direct transition from elastic deformation to fracture), as indicated by the tensile curves. Despite the low austenite fraction, a larger portion of the strain is accommodated by the γ phase, as reflected by its significantly higher KAM value (1.59°) compared to the markedly lower KAM of 0.12° observed in the α phase (Figure S10, Supporting Information). This very low KAM suggests that dislocation plasticity is nearly absent in the B2‐containing ferrite. This means the rigid ferrite strongly limits austenite deformation, impeding slip transfer across the phase boundary and intensifying dislocation pile‐up.^[^ ^68^ ^]^ Consequently, cracks nucleate at the interface and propagate into both the ferrite and the austenite, ultimately causing premature failure.

## 3 Discussion

**Figure 6:**
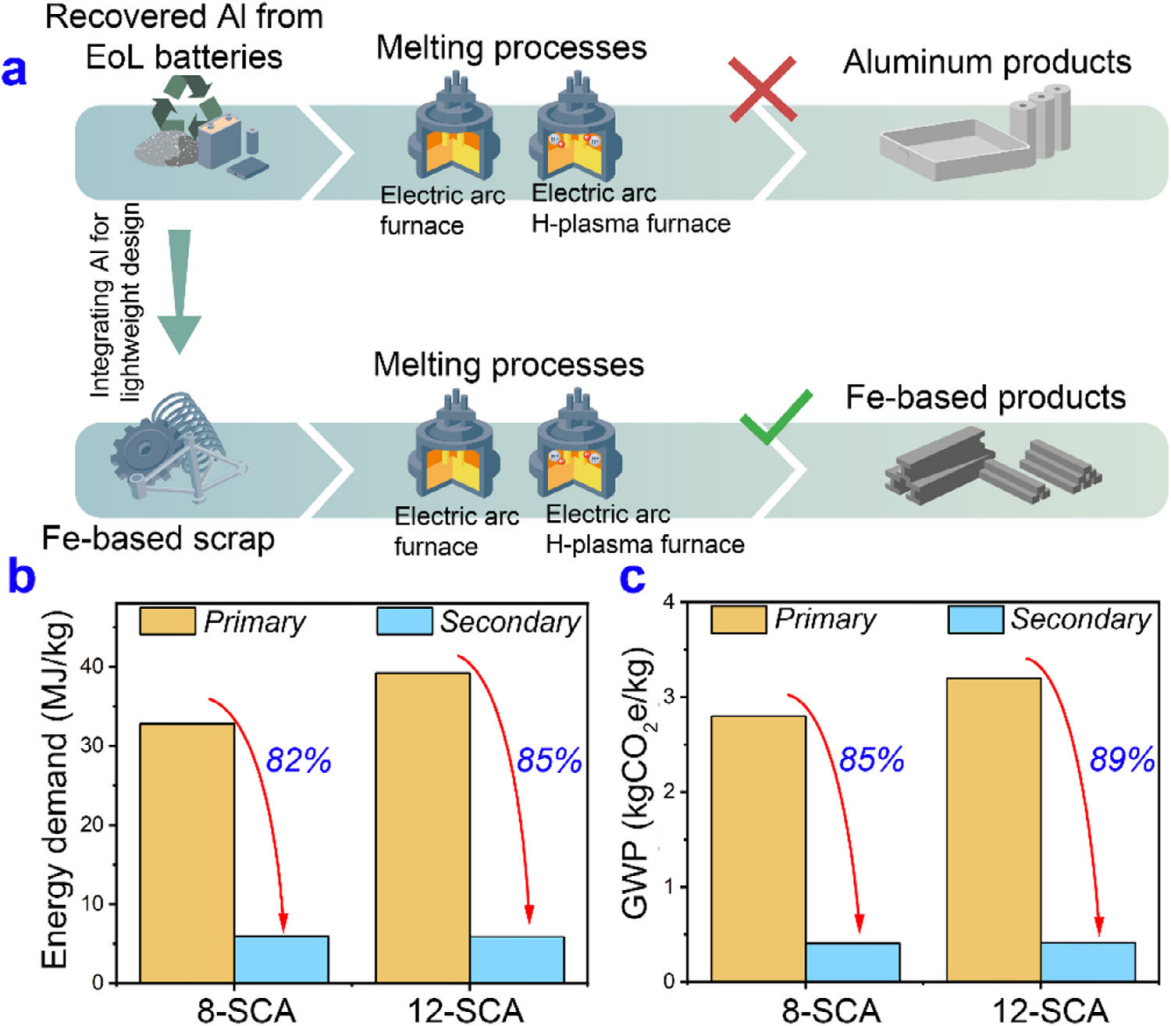
Sustainable benefits of recycling dissimilar alloys. a) Schematic illustration of the Al scrap integration with Fe‐based scraps. b) Energy demand, and c) GWP for primary and secondary synthesis for the 8‐SCA and 12‐SCA samples.

Our initial approach involved plasma remelting of Al scrap sourced from EoL batteries; however, this route was unsuccessful (Figure S2, Supporting Information). The presence of NMC oxide particles and other impurity elements (Si, Fe, Cu) limited solubility during melting, leading to the formation of coarse alumina inclusions, which compromised direct reuse. Overcoming the strong thermodynamic stability of these oxides would require energy inputs nearly equivalent to primary Al production. Using virgin Al alongside scrap Al can enable closed‐loop recycling; however, it conflicts with sustainability principles because of the high energy consumption and costs linked to primary Al production. Here, we have demonstrated an alternative recycling approach, converting Al from battery scrap into a valuable alloying addition for Fe‐based systems. This strategy, aligned with growing scrap volumes,^[^ ^69^ ^]^ promotes a shift from traditional in‐class to dissimilar alloy recycling, thereby enhancing overall sustainability. As shown in **Figure** **6**a, the significant contamination of Al scrap from EoL EV batteries prevents its direct remelting into Al products. However, it can be repurposed as a valuable alloying element in Fe‐based alloys, contributing to density reduction and validating the viability of integrating battery‐derived Al scrap with Fe‐based scrap streams.

Although the annual production of Al (92 Mt per year) is nowhere near that of steel (1850 Mt per year), its CO_2_ emissions per ton of material produced are significantly higher.^[^ ^6^ ^]^ This is because producing Al and steel from primary ores consumes roughly 180 and 20 MJ kg^−1^ of energy, resulting in ≈12 and ≈2 t CO_2_ emissions per tonne of metal, respectively.^[^ ^5^, ^25^ ^]^ In contrast, remelting scrap can lower these energy requirements by up to 90% for Al and 75% for steel, thereby substantially mitigating their carbon footprints. In lightweight steel production, Al is added to decrease the overall weight while maintaining or enhancing strength. In this study, the two alloys were designed by adding 8 wt.% and 12 wt.% Al scrap to Fe‐based scrap. Figure 6b,c shows the energy and CO_2_ savings for the case scenarios examined in this work, considering only Fe and Al as the alloy components. For a Fe‐8Al (wt.%) steel composition, this results in a reduction in energy demand from 32.8 MJ kg^−1^ (ore‐based) to 5.92 MJ kg^−1^ (scrap‐based), which is an 82% decrease in specific energy consumption. Similarly, transitioning from primary to secondary production achieves about an 85% energy savings for a Fe‐12Al (wt.%) steel. In terms of global warming potential (GWP), dissimilar alloy recycling results in an 85% and 89% reduction in CO_2_ emissions for Fe‐8Al (wt.%) and Fe‐12Al (wt.%) steels, respectively. Such substantial reductions underscore the potential of this approach to contribute to the decarbonization of ferrous metallurgy, not only by lowering direct process‐related CO_2_ emissions but also by lowering the upstream carbon footprint associated with raw material extraction. However, since recycling is plagued by contamination issues, remelting for direct reuse sometimes becomes impossible, and this calls for primary metal dilution to minimize the impact of impurities.^[^ ^69^ ^]^

Despite the overall feasibility of the co‐recycling process, the effects from the integration of impurities or tramp elements from the Al‐ and Fe‐based scrap should also be considered. Cu, known to promote hot shortness during thermomechanical processing, is the most critical impurity in steel recycling.^[^ ^70^ ^]^ In the present study, no signs of hot shortness were observed, even though Cu was uniformly distributed in the austenite (≈0.2 at.%) and locally enriched up to ≈0.7 at.% within the B2 precipitates (Figures 3 and 4; Figure S6, Supporting Information). Si, also a common impurity encountered during steel recycling, has a strong affinity for oxygen and primarily functions as a deoxidizer; however, the trace amounts that remain in the solidified microstructure tend to segregate at grain and phase boundaries (Figure 4), slightly weakening interfacial cohesion.^[^ ^50^ ^]^ This suggests that the impurity concentrations in the recycled alloys remain within a tolerable range for the proposed recycling route.

According to the phase diagrams (Figure S1, Supporting Information), an array of microstructures can be obtained by selecting specific thermomechanical processing routes. We observe that the 8‐SCA and 12‐SCA samples are austenite‐based duplex γ+α Fe‐8Al and ferrite‐based duplex α+γ Fe‐12Al steels, respectively. The ferritic chemical environment is particularly conducive to the homogeneous nucleation of B2 particles within the α phase, with both particle size and number density increasing as the Al content rises (Figure 3; Figure S6, Supporting Information). Notably, the formation of ordered B2 precipitates within ferrite is unavoidable regardless of heat treatment; a phenomenon previously observed in some steels due to the amounts of Al and Si.^[^ ^50^, ^71^ ^]^ The distribution and composition of these precipitates depend sensitively on Al content (Figures 3 and 4; Figure S6, Supporting Information), influencing the balance between strength and ductility, especially since the confinement of the B2 precipitates in the ferrite phase generates stress concentrations that induce microcrack formation.^[^ ^27^ ^]^ As such, the 12‐SCA sample with the coarser B2 number density and higher ferrite fraction suffers severe embrittlement. Thus, reducing the ferrite fraction and controlling the nanoscale B2 ordering through alloy design and thermomechanical treatment becomes an optimum recipe for achieving a better strength‐ductility combination for the recycling approach.

From an implementation standpoint, the process of incorporating Al recovered from EoL batteries into Fe‐based alloys is fully compatible with EAF and induction melting operations in secondary steelmaking. However, the successful industrial realization of this approach depends on the battery chemistry (e.g., NMC, LFP, or NCA), which determines the associated residual elements from the recovered Al. This challenge can be mitigated through preprocessing, feedstock homogenization, and adaptive refining strategies, ensuring consistent alloy quality.^[^ ^17^ ^]^ Similarly, the Fe‐scrap variations should have tolerable limits for the main deleterious impurities like Cu, S, and P to allow for adaptability.^[^ ^25^ ^]^ Overall, the results demonstrate that the integration of battery‐derived Al into Fe‐based alloys is not only technically feasible and scalable, but also represents a sustainable and economically advantageous pathway toward recycle‐friendly alloy design.

## 4 Conclusion

This work establishes a viable, sustainable approach for utilizing aluminum recovered from EoL EV batteries by integrating it as an alloying element in Fe‐based alloys rather than re‐refining it to battery‐grade purity. By embracing “dissimilar alloy recycling,” the challenges posed by contamination and limited solubility are transformed into opportunities for density reduction and tailored microstructure design. Experimental results demonstrate that controlled Al‐additions and thermomechanical processing enable the formation of desirable duplex austenite/ferrite microstructures, while careful phase fraction control mitigates embrittlement from B2 precipitates. These results demonstrate that battery‐derived Al scrap, considered too impure for reuse, can be reimagined as a strategic alloying element in Fe‐based alloys—turning contamination into a design advantage for strong, ductile, and lightweight materials. The proposed route not only conserves resources and reduces emissions but also provides a scalable pathway for integrating heterogeneous scrap streams into advanced alloy production.

## 5 Experimental Section

### Wet Chemical Analysis

Wet chemical analysis was performed on the Al scrap and Fe‐based alloy scrap using an iCAP‐6300 Duo Inductively Coupled Plasma (ICP) analyzer. A weighed sample was placed in a Teflon vessel, and 3 mL of HCl and 1 mL of HNO_3_ were added. The vessel was then placed in an UltraWave system where the temperature was increased from 0 to 270 °C and the pressure was increased to 140 bar over 15 min, followed by a holding period of 10 min. After the process, the sample was diluted with DI water to a final volume of 50 mL for subsequent analysis. Details of the chemical compositions of the scrap materials are shown in Table 1. The chemical compositions of the scrap‐based alloys (SCAs) were also analyzed similarly, and the results are also summarized in Table 1.

### Reduction Experiment

Al (5 g) scrap samples were compacted and processed in a conventional arc‐melting furnace. The chamber was filled with an Ar‐10%H_2_ atmosphere at 900 mbar, and an arc plasma was generated between the electrode and the scrap at 200 A. Melting and reduction occurred simultaneously during 1 min of plasma exposure, after which the arc was extinguished and the sample allowed to solidify. The chamber atmosphere was refreshed with a new Ar‐10%H_2_ mixture (< 18%H_2,_ the safety limit), and this cycle was repeated up to five times to obtain 5 min reduced samples.

### Scrap Integration

Al scrap recovered from EoL LIBs was compacted into pellets and employed as an alloying addition to Fe‐based alloy scrap sheets sourced from previous projects. Two alloy samples containing 8 wt.% and 12 wt.% Al scrap—designated “8‐SCA” and “12‐SCA”, respectively—were produced. To ensure chemical homogeneity, each batch was remelted at least four times in a vacuum induction furnace under a high‐purity Ar atmosphere. The molten ingots were cast into a copper mold with dimensions of 100 mm × 20 mm × 20 mm (length × width × thickness). Subsequent hot rolling at 1200 °C reduced the thickness by 75% to a final 5 mm thickness. The rolled plates were then homogenized at 1200 °C for 6 h and water‐quenched. Guided by equilibrium phase diagram predictions (Figure S1, Supporting Information) generated using Thermo‐Calc software (version 2024.1), the samples were annealed at 1000 °C and water‐quenched.

### Microstructure Characterizations

Bulk phase analysis was performed using an X‐ray Diffraction (XRD) Bruker D8 Advance A25. Microscale compositional and phase analyses of the samples were performed using energy‐dispersive spectroscopy (EDS) and electron backscatter diffraction (EBSD) on a Zeiss Merlin scanning electron microscope (SEM) equipped with EDAX software. The EBSD datasets were processed using OIM Analysis software (Version 9). Atom probe tomography (APT) specimens were prepared from bulk material using a FEI Helios Dual‐Beam Xe‐plasma focused ion beam (FIB). A Cameca LEAP 5000XR in laser‐pulsed mode was employed for APT measurements at 60 K, 125 kHz pulse rate, and 60 pJ pulse energy. The resulting datasets were analyzed using AP Suite software (Version 6.3.2.128).

### Room‐Temperature Tensile Tests

Flat, dog‐bone–shaped tensile specimens (total length: 54 mm; gauge length: 30 mm; gauge width: 5 mm; thickness: 2 mm) were fabricated via electrical discharge machining along the rolling direction. Uniaxial tensile tests were performed at room temperature under a strain rate of 1 × 10^−3^s^−1^. Each test was repeated three times per composition to ensure reproducibility.

## Conflict of Interest

The authors declare no conflict of interest.

## Supporting information



Supporting Information

## Data Availability

The data that support the findings of this study are available from the corresponding author upon reasonable request.
