# Supplementary material for: Where Can Aluminum Go When Batteries Die?
Source: Adv Sci (Weinh). 2025 Nov 16;13(7):e18482. doi: 10.1002/advs.202518482 (PMC12866715; doi:10.1002/advs.202518482)
Supplement: Supplementary file 1 — Supporting Information [file ADVS-13-e18482-s001.docx]

Supplementary information for

**Where can aluminum go when batteries die?**

Raymond Kwesi Nutor^1^*, Waleed Mohammed^1^, Se-Ho Kim^1,2^, Baptiste Gault^1,3^*

^1^ Max-Planck-Institute for Sustainable Materials, 40237, Düsseldorf, Germany.

^2^ Department of Materials Science and Engineering, Korea University, Seoul, 02841, Republic of Korea.

^3^ Department of Materials, Imperial College London, London, SW7 2AZ, UK.

*Corresponding authors’ e-mails: r.nutor@mpie.de, b.gault@mpie.de.

**This PDF file includes:**

Figs. S1 to S10


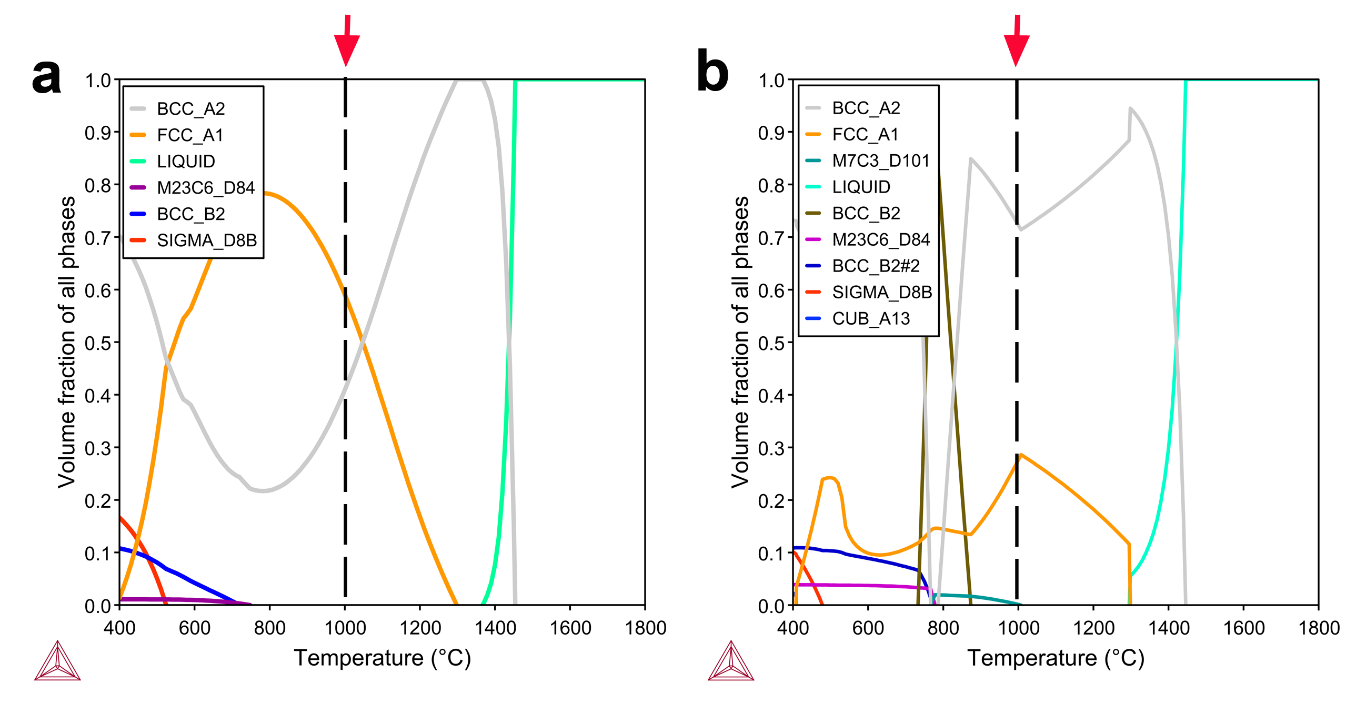


**Fig. S1. Thermodynamic phase diagrams for recycled alloy samples.** Equilibrium phase diagrams for **a** 8-SCA and **b** 12-SCA samples.


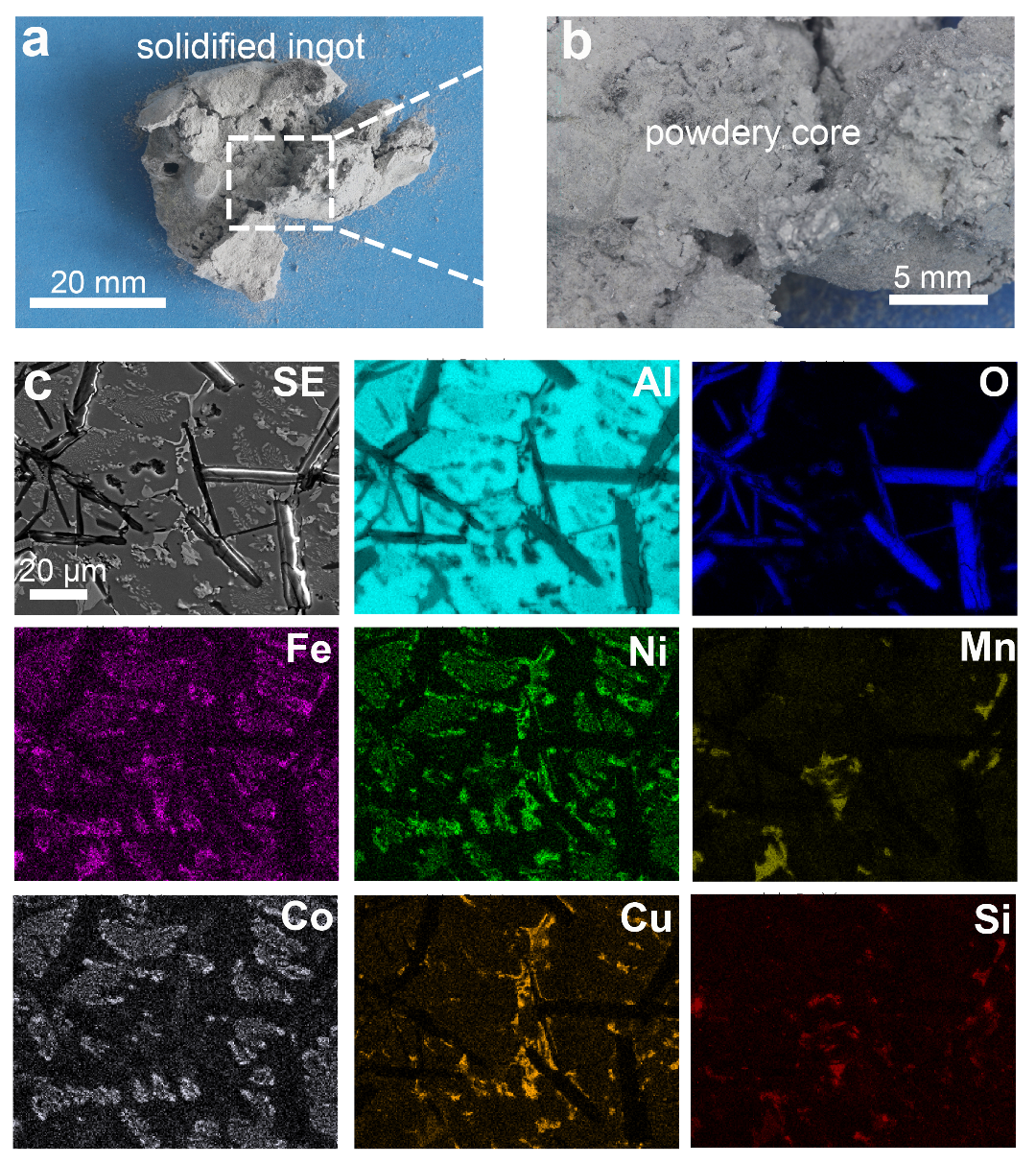


**Fig. S2. Morphology and microstructure of remelted Al ingot. a-b** Photographs of the solidified ingot showing porosities, which are likely gas entrapments. **c** SEM image of the Al ingot and the correlative EDS maps showing the distribution of impurities.


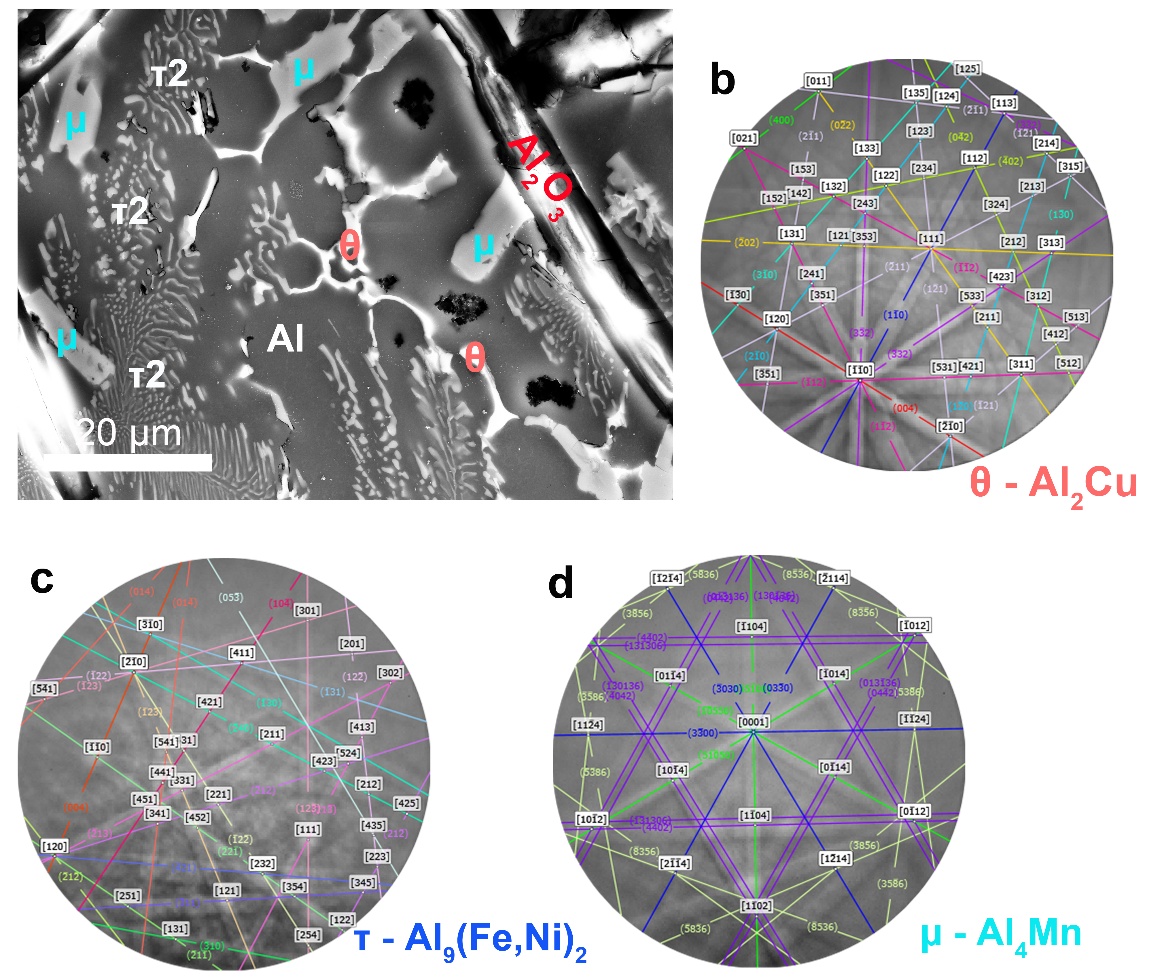


**Fig. S3. Morphology of intermetallic and oxide phases in remelted Al ingot. a-b** SEM image of the Al ingot showing the different intermetallics and oxides. Representative indexed EBSD Kikuchi patterns of the **b** tetragonal θ-Al_2_Cu, **c** hexagonal τ2-Al_9_Fe_2_Ni, and **d** hexagonal µ-Al_4_Mn phases, respectively.


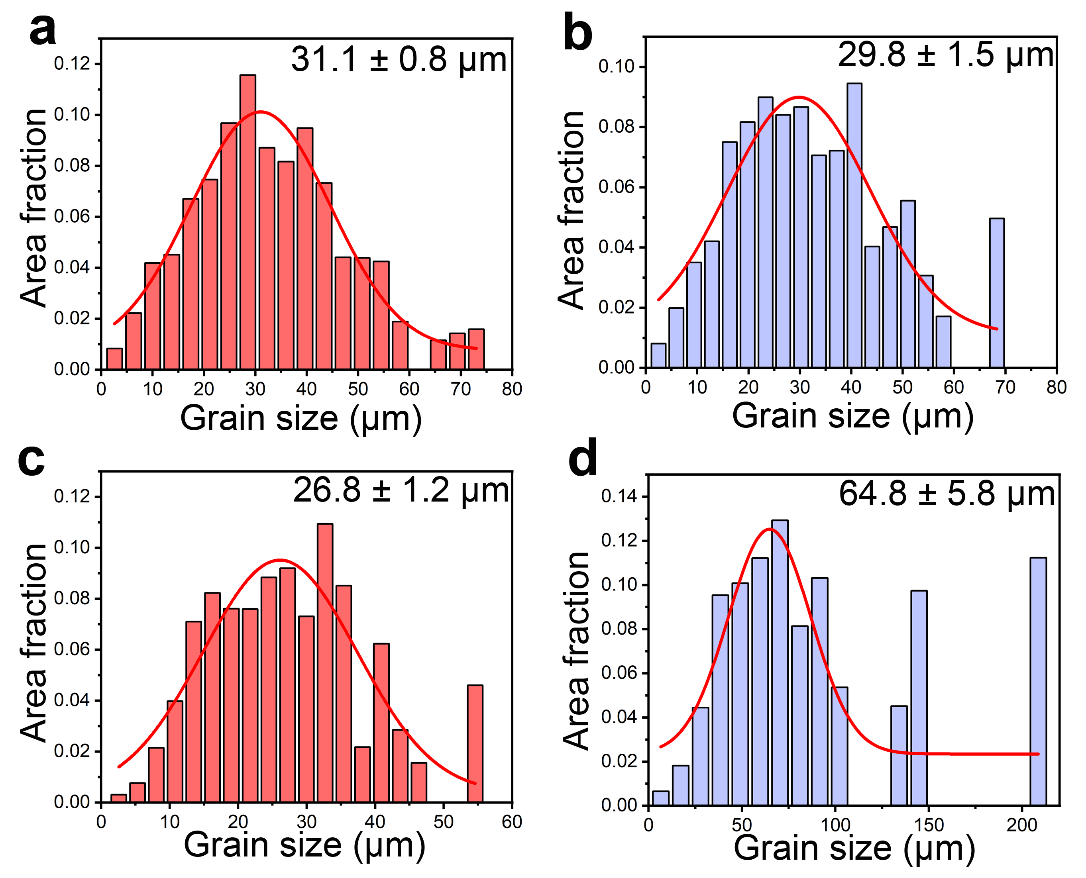


**Fig. S4. Grain size distribution of constituent phases estimated from EBSD maps of the SCA samples.** **a, b** Average grain sizes of the γ and α phases in the 8-SCA sample. **c, d** Average grain sizes of the γ and α phases in the 12-SCA sample.


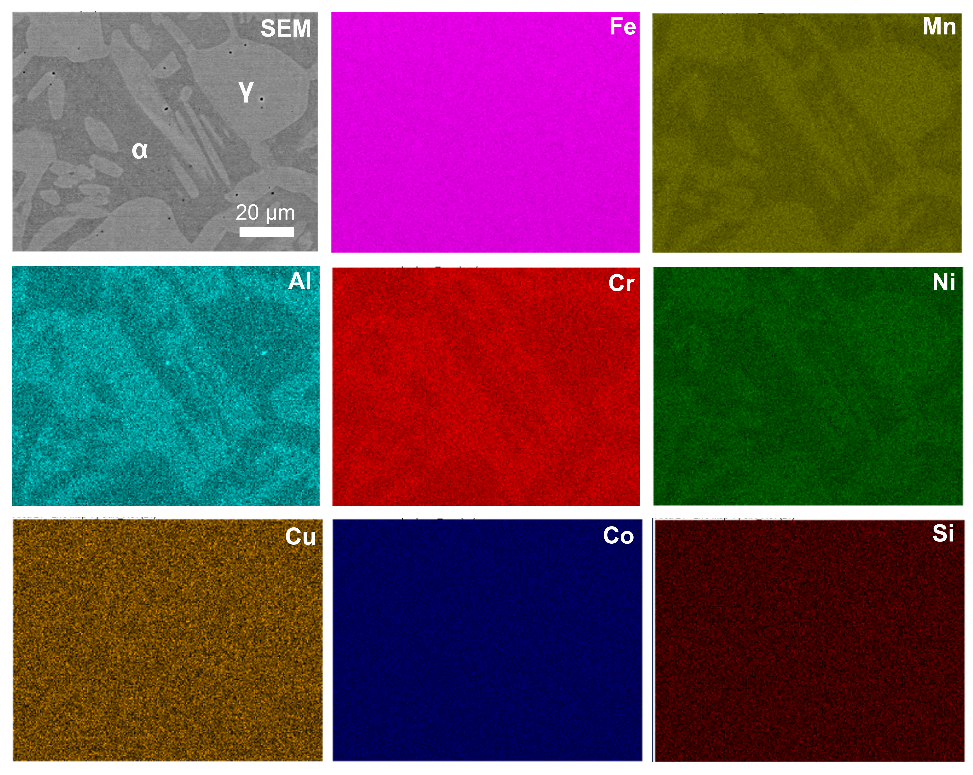


**Fig. S5. SEM-EDS elemental maps for the 8-SCA sample.** Elemental maps of constituent elements in the studied 8-SCA sample. Mn, Cr, Ni, and Al show notable partitioning in the different phases, while Fe, Cu, Co, and Si are uniformly distributed in the austenite and ferrite phases.


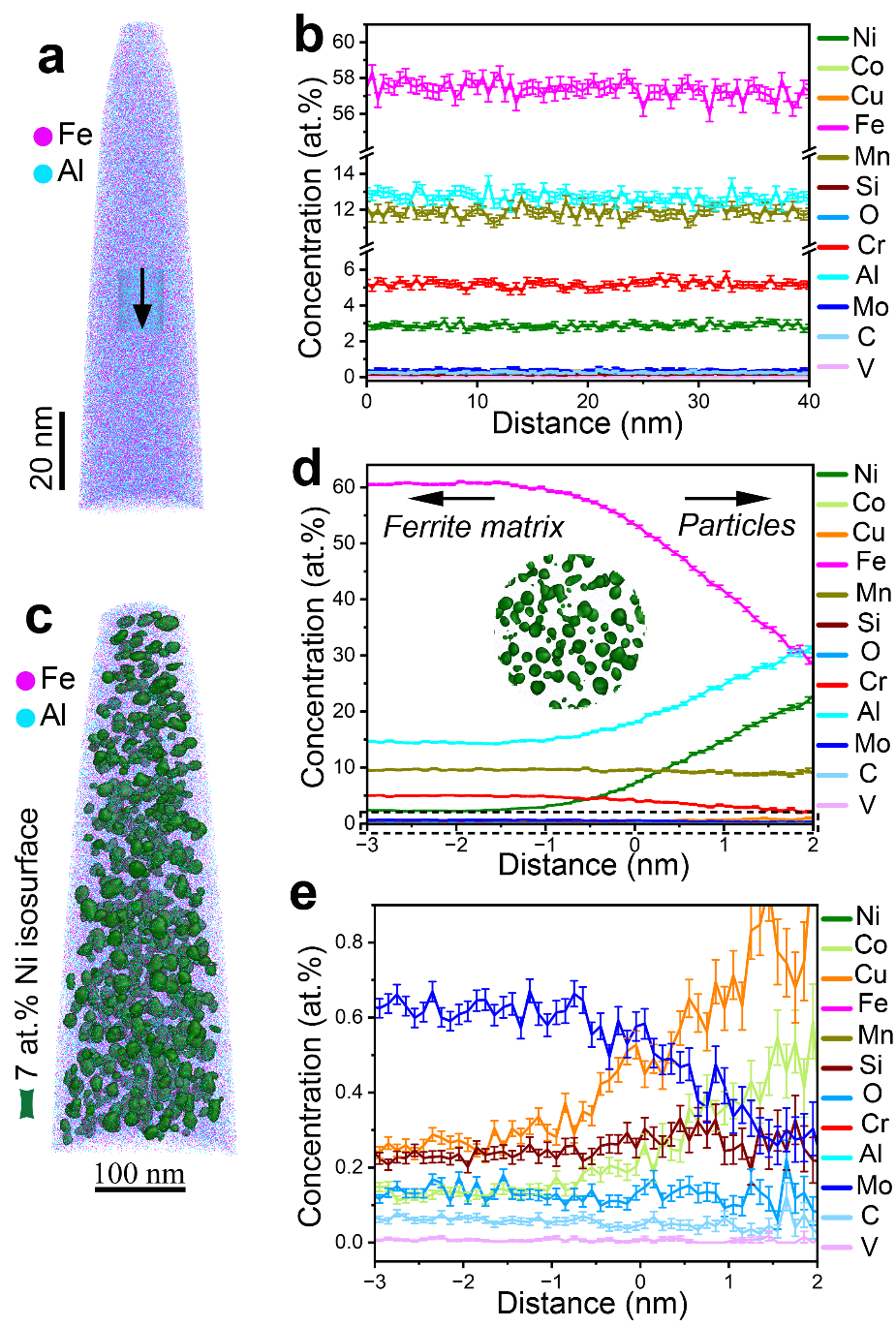


**Fig. S6. Elemental distribution in the γ** **and α phases in the recycled 12-SCA sample. a** 3D APT reconstruction of the grain interior within the γ phase. **b** 1D concentration profile of constituent elements across the γ grain interior, extracted along the cylindrical analysis volume. **c** 3D APT reconstruction highlighting a 7 at.% Ni isosurface. **d** 1D proximity histogram demonstrating the compositional changes between the α matrix and particles, with the inset revealing nanoparticle distribution within the α phase. **e** Corresponding magnified view of the region marked by the dashed rectangle in **d**.


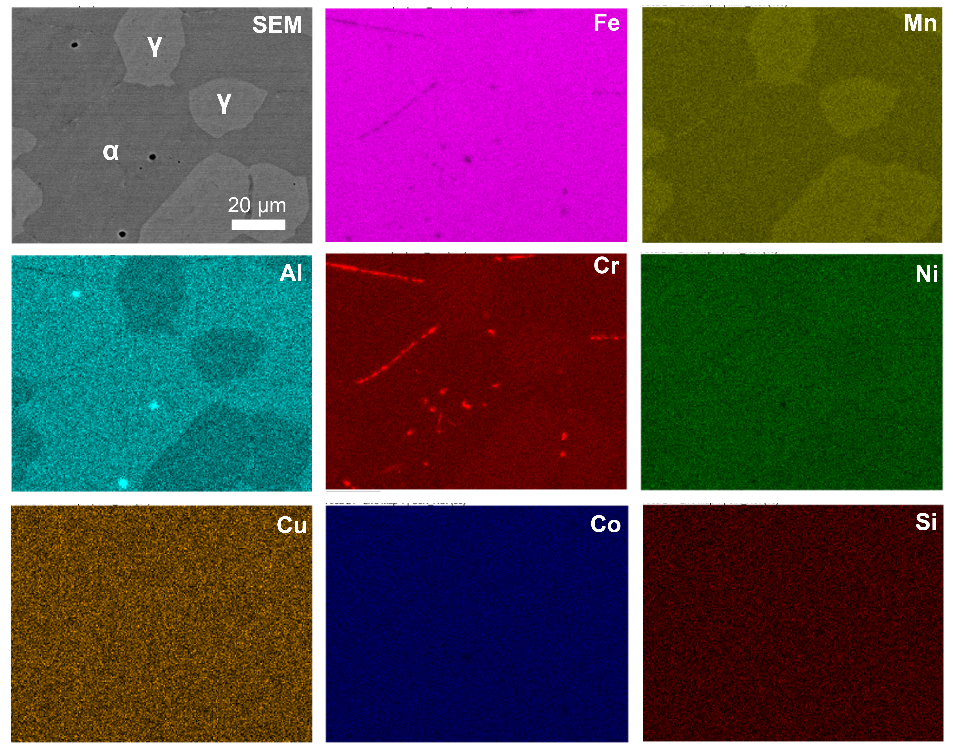


**Fig. S7. SEM-EDS elemental maps for the 12-SCA sample.** Elemental maps of constituent elements in the studied 12-SCA sample. Mn, Cr, Ni, and Al show notable partitioning in the different phases, while Fe, Cu, Co, and Si are uniformly distributed in the austenite and ferrite phases. Cr segregation is observed within the ferrite grains and at grain boundaries.


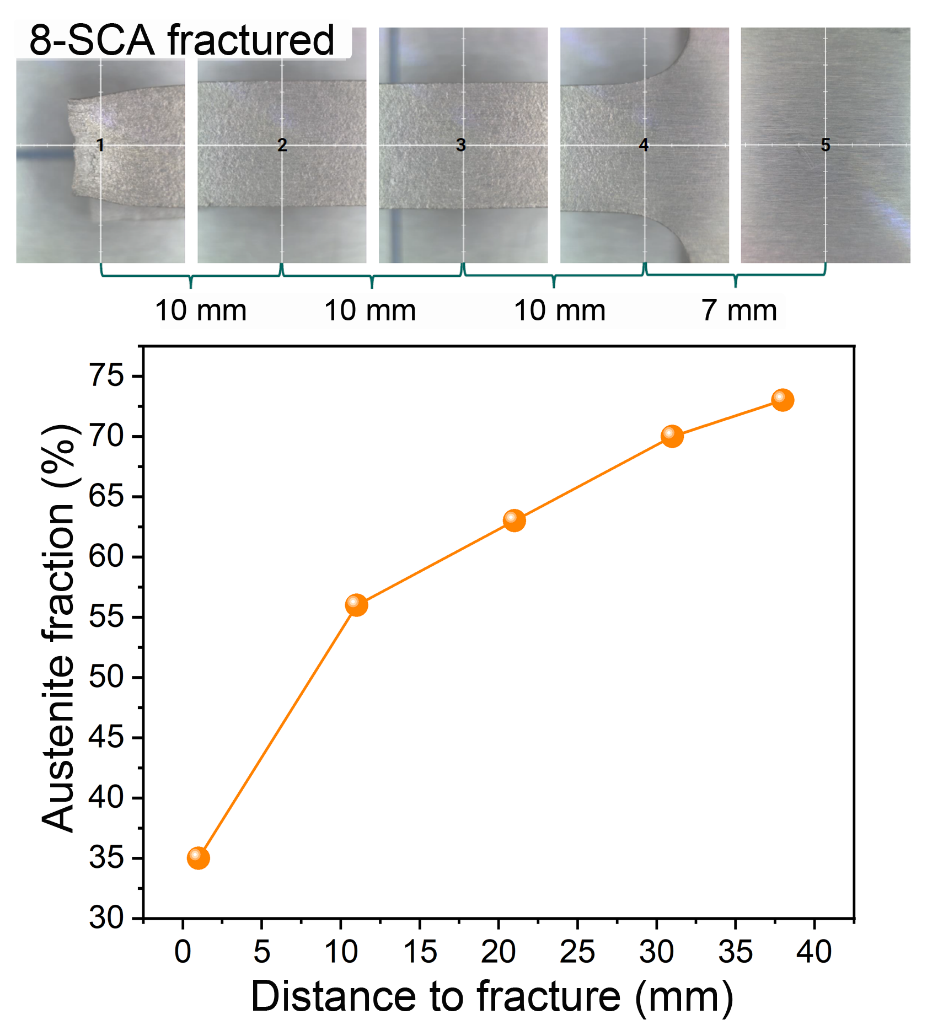


**Fig. S8. Phase evolution at different regions of the tensile-fractured 8-SCA sample.** XRD measurements were performed at set distances from the fracture region to detect the changes in the austenite fraction during deformation.


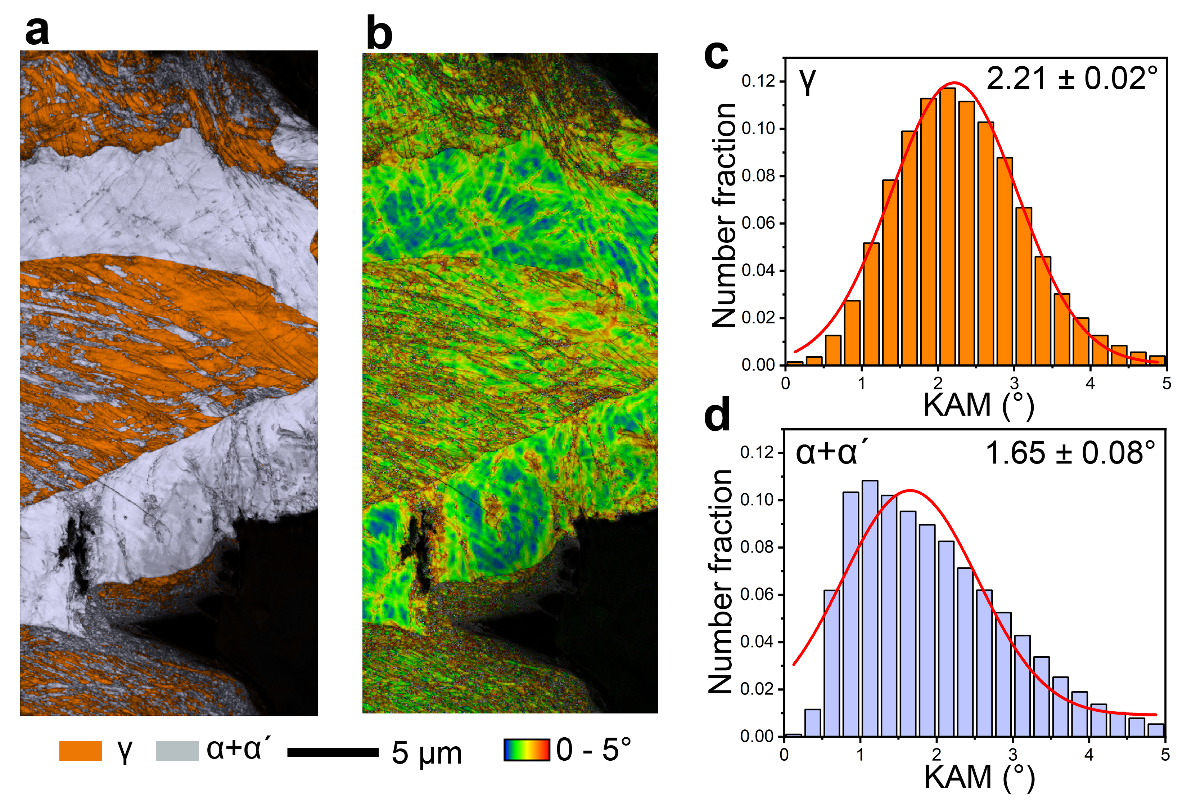


**Fig. S9. Deformation microstructure of the tensile fractured 8-SCA sample. a** EBSD IQ + phase map, and **b** corresponding kernel average misorientation (KAM) map at the fracture region of the 8-SCA sample. Average KAM distribution of the **c** γ and **d** α+α′ phases.


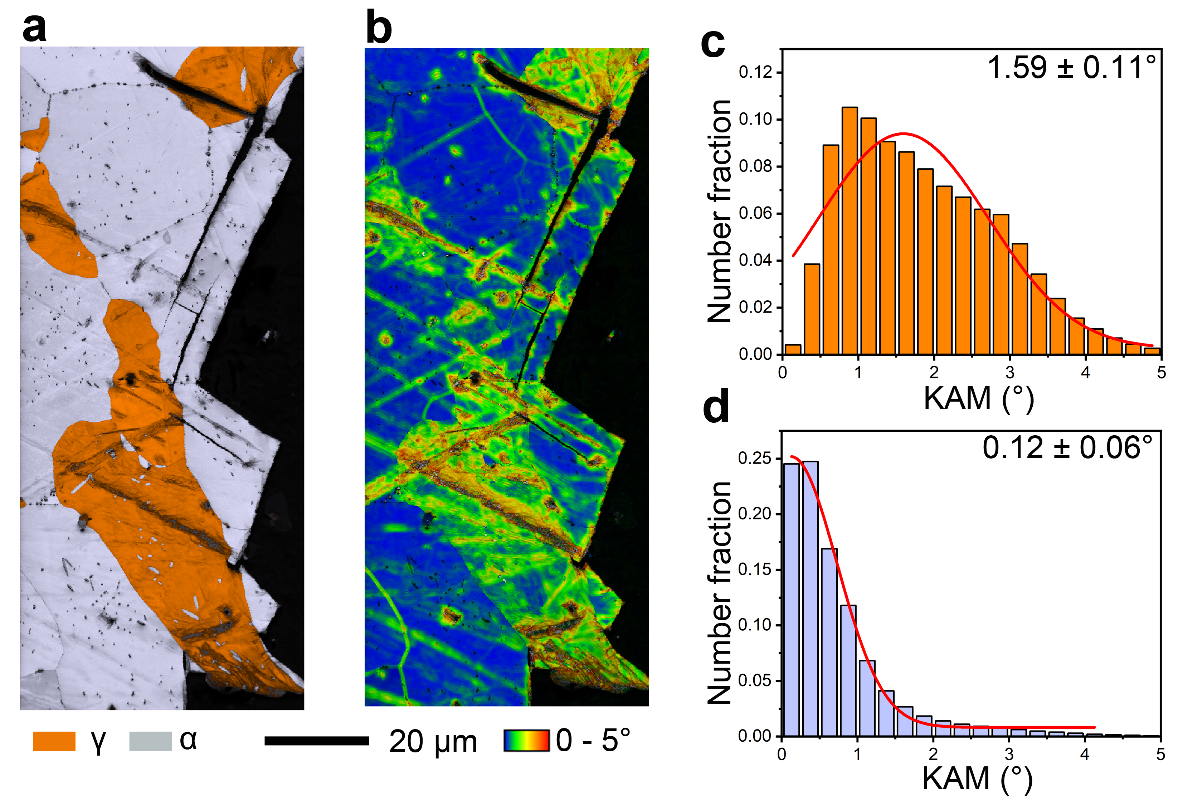


**Fig. S10. Deformation microstructure of the tensile fractured 12-SCA sample. a** EBSD IQ + phase map, and **b** corresponding kernel average misorientation (KAM) map at the fracture region of the 12-SCA sample. Average KAM distribution of the **c** γ and **d** α phases.
